# Vanadium Stress Alters Sweet Potato (*Ipomoea batatas* L.) Growth, ROS Accumulation, Antioxidant Defense System, Stomatal Traits, and Vanadium Uptake

**DOI:** 10.3390/antiox11122407

**Published:** 2022-12-05

**Authors:** Sunjeet Kumar, Mengzhao Wang, Yonghua Liu, Zhixin Zhu, Shah Fahad, Abdul Qayyum, Guopeng Zhu

**Affiliations:** 1 Key Laboratory for Quality Regulation of Tropical Horticultural Crops of Hainan Province, School of Horticulture, Hainan University, Haikou 570228, China; 2 Sanya Nanfan Research Institute, Hainan University, Sanya 572025, China; 3 Department of Agronomy, Abdul Wali Khan University Mardan, Mardan 23200, Pakistan; 4 Department of Agronomy, The University of Haripur, Haripur 22620, Pakistan

**Keywords:** vanadium stress, sweet potato, oxidative damage, antioxidant defense system, photosynthesis, stomatal traits

## Abstract

Vanadium (V) is a heavy metal found in trace amounts in many plants and widely distributed in the soil. This study investigated the effects of vanadium concentrations on sweet potato growth, biomass, root morphology, photosynthesis, photosynthetic assimilation, antioxidant defense system, stomatal traits, and V accumulation. Sweet potato plants were grown hydroponically and treated with five levels of V (0, 10, 25, 50, and 75 mg L^−1^). After 7 days of treatment, V content at low concentration (10 mg L^−1^) enhanced the plant growth and biomass; in contrast, drastic effects were observed at 25, 50, and 75 mg L^−1^. Higher V concentrations negatively affect the relative water content, photosynthetic assimilation, photosynthesis, and root growth and reduce tolerance indices. The stomatal traits of sweet potato, such as stomatal length, width, pore length, and pore width, were also decreased under higher V application. Furthermore, V concentration and uptake in the roots were higher than in the shoots. In the same way, reactive oxygen species (ROS) production (hydrogen peroxide), lipid peroxidation (malondialdehyde), osmolytes, glutathione, and enzymes (catalase and superoxide dismutase) activities were increased significantly under V stress. In conclusion, V at a low level (10 mg L^−1^) enhanced sweet potato growth, and a higher level of V treatment (25, 50, and 75 mg L^−1^) had a deleterious impact on the growth, physiology, and biochemical mechanisms, as well as stomatal traits of sweet potato.

## 1. Introduction

Vanadium (V) is world’s fifth most abundant transition element and deposit, mainly in China, the USA, Russia, and South Africa [1,2]. China is the leading producer and consumer of V, with 57% of V production globally. Around 26.5% of V-contaminated soil is present in southwest China [3,4]. V is widely distributed and mobilized in the surrounding environments by several natural events and anthropogenic activities, including weathering of parental rocks, redox processes, leaching, fertilizers usage, combustion, and industrial wastes, which as a result, contaminates the water, soil, and atmosphere [4,5]. V is deposited naturally in the soil in different mineral forms, and the average concentration of V ranges from 3 to 310 mg kg^−1^ in soil. The average V concentration in fresh, ground, and drinkable water is 0.5 µg L^−1^, with peak concentrations in volcanic areas reaching 127.4 µg L^−1^ [6]. Accumulating V in the natural habitat increases soil and water contamination, ultimately affecting human health by causing nausea, vomiting, dizziness, and more seriously, leading to kidney damage [7]. Different studies have reported that V has detrimental effects on the growth and development of plants [6,8]. V toxicity, bioaccumulation, and bioavailability rely on the oxidation state of vanadium. Vanadate is the toxic chemical compound of V, and at high concentrations hinders phenotypic, physiological, and biochemical processes of plants, eventually obstructing plant growth and yield [8,9,10].

V can be easily uptake by the plants from the soil; however, the effect is dependent on the V content in the soil. A low level of V treatment enhances plant growth, photosynthesis, and gas exchange elements [11,12]. Furthermore, Altaf et al. reported that 35 mg L^−1^ V treatment caused a drastic decline in the biomass of rice plants [13]. Similarly, Chen et al. exposed *Ipomoea aquatica* to 0–2.50 mg L^−1^ V treatments, and they concluded that concentration-dependent effects of V can be observed for the physiological properties and the plants may adapt to the toxicity of V [12]. Another study demonstrated that tobacco has good vanadium tolerance at < 2.0 mg L^−1^ [10]. A high level of V treatment negatively influences plant growth and yield by increasing reactive oxygen species (ROS) production, hindering lipid membranes, antioxidant enzyme activity, the metabolic process, and gene expression [14,15]. The plants overcome oxidative stress and scavenge ROS by enhancing the production of osmolytes, antioxidants, and stimulating the activities of the antioxidant enzymes [16,17].

Previous studies highlighted the V effect on plant physiology by reducing or altering the photosynthetic rate, shoot and root survival, and leaf chlorosis [8,18]. Photosynthesis is a vital plant metabolic process that increases carbon absorption and yield production; however, its production and fixation can be strongly hindered under heavy metal stress [13,19]. Different studies reported a significant reduction in photosynthetic pigments and gas exchange elements in pepper and watermelon under heavy metals, including vanadium, nickel, and selenium stress [11,20]. Photosynthetic pigments can be impaired by obstructing electron transport and hindering the membrane integrity of the chloroplast. Similarly, plant roots are the essential organ that interacts with and absorbs metal and other components of the soil. Roots are considered the first line of defense and give structural support to the plant against heavy metal toxicity. It helps to defend itself by minimizing the absorption of unnecessary metal [21]. Previous studies also depicted that a higher level of V supplementation significantly reduced root architecture in several plants [11,13,22]. Different plants react differently to V stress, so more research is needed to determine the best concentration of V as a biostimulant in different cultivars of the crops.

Sweet potato (*Ipomoea batatas* L.) has been used as a major source of carbohydrate in many countries around the word, especially Asia and Africa [23]. Additionally, sweet potato roots are used for biofortification and bioethanol production. Thus, the production of sweet potato can be an integral part of food security in the future [24,25]. Moreover, sweet potato stems and leaves can be used as a vegetable for humans and animal feed [26,27]. In addition, sweet potato leaves are rich in protein, iron, calcium, fiber, carotenoids, vitamins, and total polyphenols and possess medicinal properties [28,29]. Sweet potato can severely affect productivity and quality under heavy metal stresses [23,30]. Several studies found that trace amounts of certain elements may stimulate the growth and production of horticultural crops [11,31]. Conversely, V application showed deleterious effects on the growth of different plant species by obstructing their antioxidant defense system [13,20]. However, the physiological and biochemical response of the sweet potato plant under V stress has not been well-studied and requires further research. Therefore, it is essential to explore the influence of V in sweet potato plants, primarily to determine the toxicity level of V for sweet potato. We designed this study to investigate V’s effects on sweet potato plants and identify the toxic level of V. Our objective was to determine the effects of V on sweet potato growth, biomass, root morphology, photosynthesis, photosynthetic assimilation, antioxidant defense, stomatal traits, and V uptake. 

## 2. Methodology

### 2.1. Seedling Collection, Growth Conditions, and Experimental Design

In this experiment, we used the “Haida HD7791” sweet potato cultivar. For the disinfection of sweet potato cuttings, 1 g L^−1^ carbendazim was used for 5–8 min. Afterwards, the sweet potato cuttings were kept in Ro water until the roots appeared. For the acclimation, the cuttings of sweet potato were grown in half Hoagland media (pH 5.8 ± 0.2). A hydroponic experiment in a controlled environment (25–27 °C for 16 h of photoperiod) was conducted to assess V’s effect on sweet potato plants. For proper nutrient availability, the nutrient media were replaced after every 5 to 6 days. Subsequently, healthy and uniform seedlings were distributed among the five treatment groups. V (0, 10, 25, 50, and 75 mg/L) was applied as ammonium metavanadate (NH_4_VO_3_). For the analysis of morphological and physiological measurements, samples were collected after 7 days of treatment with V or normal growth conditions and immediately transferred into a liquid nitrogen tank. 

### 2.2. Growth Variables

Three independent seedlings were used to measure plant height (PH), the leaf area (LA), number of leaves (LN), and fresh and dry weights of the roots and shoots. A portable laser leaf area meter (CI-202) was used to measure the LA (topmost leaves). A ruler was used to measure the height of the seedlings. The seedlings were cut, and the fresh weight of the shoot and root was recorded. For recording the dry weight (DW), the samples were first dried at 105 °C for 30 min and then kept for drying at 70 °C for 3 days [32]. The plant’s shoot DW susceptibility index (SDSI) was calculated as follows:

(1) $$\mathrm{SDSI}=\frac{\mathrm{Shoot}\;\mathrm{DW}\;\left(\mathrm{stressed}\;\mathrm{plants}\right)}{\mathrm{Shoot}\;\mathrm{DW}\;\left(\mathrm{controlled}\;\mathrm{plants}\right)}\times 100$$

Similarly, the following formula was used to calculate the plant’s RDSI;

(2) $$\mathrm{RDSI}=\frac{\mathrm{Root}\;\mathrm{DW}\;\left(\mathrm{stressed}\;\mathrm{plants}\right)}{\mathrm{Root}\;\mathrm{DW}\;\left(\mathrm{controlled}\;\mathrm{plants}\right)}\times 100$$

### 2.3. Relative Water Content Analysis

After recording the FW, the sweet potato leaves were immersed in ddH_2_O for four hours. After 4 h the leaves were weighed to determine the turgor weight (TW). To determine the dry weight (DW), the leaves were oven-dried for one day at 70 °C [32]. Finally, the RWC was measured using the following formula:

RWC% = [(FW − DW)/(TW − DW)] × 100(3)

### 2.4. Root Morphology

After surface rinsing, the roots were washed with ddH_2_O and scanned with the Imagery Screen (Epson Expression 11000XL, Regent Instruments, Chemin Sainte-Foy, QC, Canada) to observe different root traits. The images obtained from the root scanner were analyzed with the WinRHIZO 2003a software program [13].

### 2.5. Gas Exchange Parameters

Gas exchange parameters of sweet potato leaves were determined for completely matured leaves utilizing a portable photosynthesis system (CIRAS-3, Hansatech Co., Amesbury, MA, USA) [13]. 

### 2.6. V Determination, Uptake, and Translocation

A super microwave-assessed digestion system (Anton Paar, Multiwave 7000, Styria, Garz, Austria) was used to digest plant samples (100 mg dry weight) with 2 mL HNO_3_, 0.5 mL H_2_O_2_, and 1 mL deionized water. We used the standard reference material (GBW10015) in triplicate for quality control and assurance (obtained from the Chinese Academy of Geological Sciences, Langfang, China). The reference CRM standard value was V(10^−6^) 0.87 ± 0.23. The working standards of V (0–200 µg/L) were made using a standard stock solution (GSB04-1759-2004, Beijing, China) containing 1000 mg/L of V. The inductively coupled plasma mass spectrometer (ICP-MS) (Perkin Elmer, NexION 5000, Waltham, MA, USA) was used to measure the V content [32]. The detection limit (DL) of standard V in solution was 0.01 μg/L (0.001522 ppb), the correlation coefficient was 0.999977, and the recovery of CRM standard V ranged from 86.5% to 93%. The subsequent formula to measure V uptake and translocation was used:

V uptake (mg) = V concentrations in the tissues × dry weight of the tissues(4)

(5) $$\mathrm{Translocation}=\frac{V\;\mathrm{concentration}\;\mathrm{in}\;\mathrm{the}\;\mathrm{plant}\;\mathrm{shoots}}{V\;\mathrm{concentration}\;\mathrm{in}\;\mathrm{the}\;\mathrm{plant}\;\mathrm{roots}}$$

### 2.7. Measurement of Photosynthetic Pigments

Leaf samples (0.1 g FW) were mixed with 80% acetone, followed by centrifugation for 15 min at 8000× *g*. A microplate reader (Infinite M200 PRO, TECAN, Männedorf, Swiss) was used for measuring the absorbance of chlorophyll a (chl a), chl b, and carotenoids (Car) at 663, 646, and 470 nm, respectively. Finally, the concentration of chlorophyll was calculated with the subsequent formula reported by Kumar et al. [16].

### 2.8. Determination of Malondialdehyde (MDA)

A kit (A003-1-1) was used to quantify MDA following the thiobarbituric acid (TBA) method. A glass homogenizer was used to properly homogenize 100 mg of fresh leaves in 900 µL of extraction buffer provided by the company. The homogenate was centrifuged at 5000× *g* for 15 min, followed by three centrifugations of 15 s each at 4000× *g*, with a 30 s interval between each centrifugation, with the final centrifugation of 3500× *g* for 10 min. After that, the supernatant was collected and mixed with the working fluid (combination of R1: clarifying agent, R2: buffering agent, and R3: color developer in the ratio of 0.1:3:1) provided by the company, then the mixture was boiled at 95 °C for 20 min. After cooling, the absorbance at 530 nm was determined using a full-wavelength microplate reader (Infinite M200 PRO, TECAN, Männedorf, Swiss) [32]. 

### 2.9. Determination of Hydrogen Peroxide, Proteins, GSH, and Antioxidant Enzymes

A homogenized sample of 500 mg of fresh leaves was centrifuged at 10,000× *g* for 15 min with 4.5 mL of 0.1 M PBS. The hydrogen peroxide (H_2_O_2_), total proteins, reduced glutathione (GSH), and antioxidant enzymes, catalase (CAT), peroxidase (POD), superoxide dismutase (SOD), and ascorbate peroxidase (APX) activities were measured using commercially available test kits purchased from Nanjing Jiancheng Bioengineering Institute, Nanjing, China and their absorbance were determined using a full-wavelength microplate reader (Infinite M200 PRO, TECAN, Männedorf, Swiss) [17,32,33].

### 2.10. Determination of Proline and Soluble Sugars

Proline was calculated by using an assay kit (A107-1-1). Fresh leaf samples were homogenized with buffer available in the kit and tested at 520 nm following the company’s protocol. Approximately 50 mg of fresh leaves were homogenized in 0.45 mL ddH_2_O for the analysis of soluble sugars. The homogenate was boiled at 95 °C for 15 min and then centrifuged at 7500× *g* for 15 min. After that, the supernatant was collected and diluted with ddH_2_O at 1:9. Using a test kit (A145-1-1), the soluble sugar content of the diluted extracts was determined at 620 nm [17,32]. 

### 2.11. Determination of Total Polyphenols and Flavonoid Content

A fresh leaf sample of 1 g was homogenized with 60% ethanol. After that, 1.25 mL of 10% Folin–Ciocalteu reagent was added to 250 µL of extract and 1 mL of sodium carbonate (0.75 g/mL). After incubating for 15 min at 45 °C, the mixture was allowed to remain at room temperature for 30 min. As a final step, the absorbance was recorded at 765 nm, and the results were presented as Gallic acid equivalents per gram (GAE/g) to quantify total polyphenols [16,34]. 

Approximately 0.25 mL of NaNO_2_ solution (0.5 g/mL) and 2 mL ddH_2_O were mixed with 0.5 mL of extract to measure flavonoids. After being retained at 25–28 °C for 5 min, 150 µL of aluminium chloride (1 g/mL), 1 mL of NaOH (1 M), and 1.2 mL of ddH_2_O were added simultaneously. As a final step, its absorbance was measured at 510 nm with Catechin (CAE) used as a standard, and its results were presented as CAE/g [16,34].

### 2.12. Scanning Electron Microscopy (SEM)

To observe the stomatal morphology, we used a published protocol [30]. To remove any debris, leaves were acetylated in 80% ethanol for two to three min. The tiny sections of leaf were prepared using s-cutting, and after that platinum was used to fix the abaxial and adaxial surfaces and sputtered using Leica Mikrosystem GmbH (ACE600) for 25 min, and finally examined under a SEM (Thermo Scientific, Verios G4 UC, Waltham, MA, USA).

### 2.13. Statistical Analysis

Three individual replications were used to obtain phenotypic, physiological, and biochemical indices. Significant differences (*p* ≤ 0.05) between means were determined using SPSS 25.0 software, and Duncan tests were applied for the means comparison, while ± represents a standard error (S.E). Figures were plotted with GraphPad Prism 7. The “ggplot2” package in R (version 3.3.4, https://CRAN.R-project.org/package=ggplot2 (accessed on 24 August 2022)) was used for principal component analysis (PCA) and Pearson correlation analysis.

## 3. Results

### 3.1. Growth Parameters, RWC, and Tolerance Index (TI)

**Figure 1:**
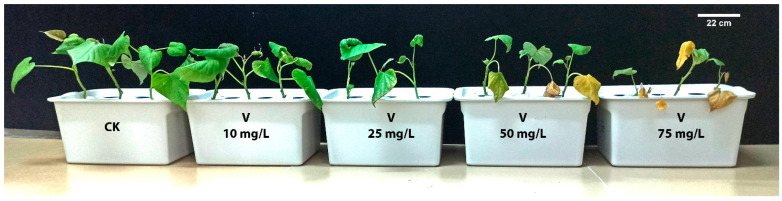
The influence of various V treatments on the growth of sweet potato.

After 7 days of V treatment, the growth traits of sweet potato were significantly influenced by the treatment of V stress (Figure 1). The phenotypic parameters, such as PH, LA, LN, shoot and root FW, DW, SDSI, and RDSI were increased at 10 mg L^−1^ V treatment. Conversely, a significant reduction in the mentioned parameters was observed at 25, 50, and 75 mg L^−1^ V treatment compared to the control (*p* < 0.05; Table 1 and Table 2). The PH (8.6%), LN (11.7%), LA (7.6%), SFW (14%), RFW (16.4%), SDW (25.5%), RDW (28.9%), root-to-shoot ratio (3.1%), SDSI (25.5%), and RDSI (30.2%) were increased at 10 mg L^−1^ V treatment than the control. In contrast, at higher concentrations, V treatment showed a negative correlation with plant growth. Furthermore, a drastic reduction in the PH (40.6%), LN (58.8%), LA (61.1%), SFW (57.8%), RFW (68.6%), SDW (62.2%), RDW (77.2%), root-to-shoot ratio (38.8%), SDSI (62.1%), and RDSI (76.3%) were observed at 75 mg L^−1^ V treatment (Table 1 and Table 2). Moreover, RWC in the leaves of sweet potato reduced significantly as the level of V treatment increased, and the utmost reduction was noted at 75 mg L^−1^ V treatment (Table 1). 

### 3.2. Root Morphology

**Figure 2:**
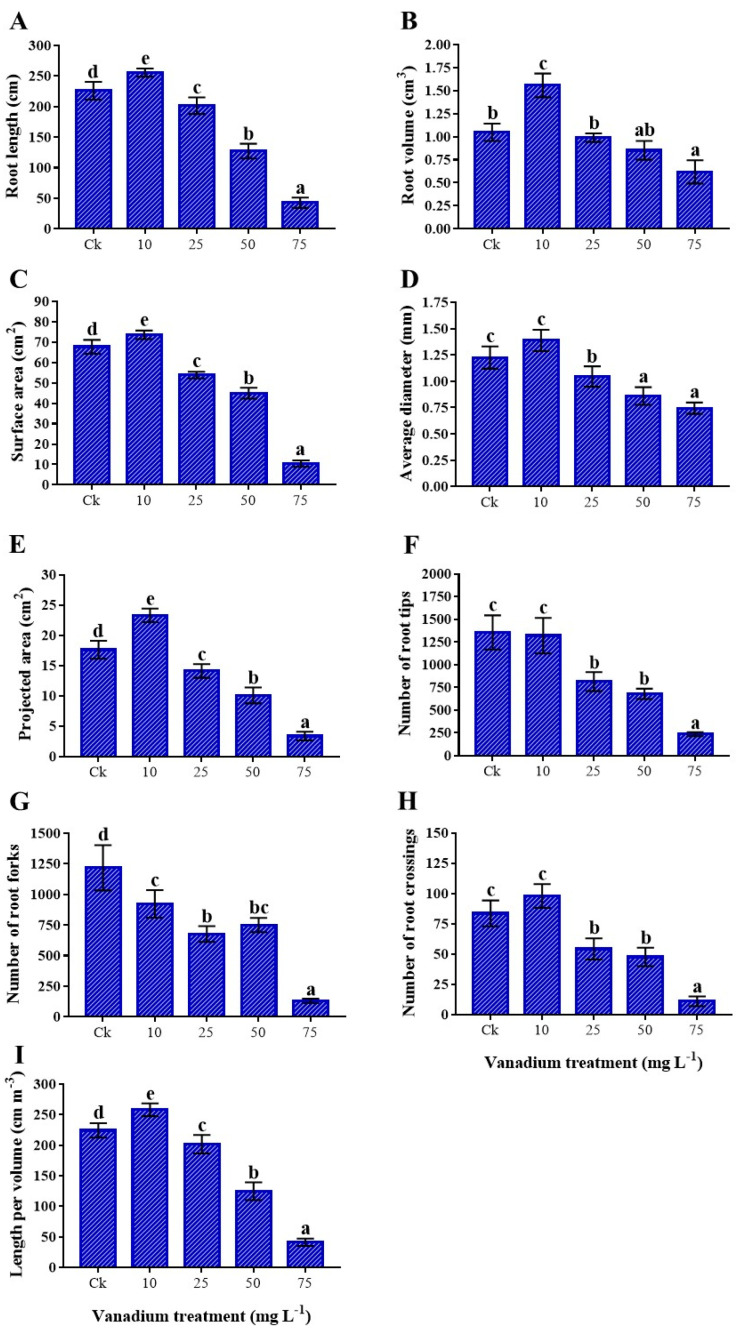
The influence of various V treatments on root morphological traits; (**A**) root length, (**B**) root volume, (**C**) surface area, (**D**) average diameter, (**E**) projected area, (**F**) number of root tips per plant, (**G**) number of root forks per plant, (**H**) number of root crossings per plant, and (**I**) root length per volume. Duncan’s test specifies a significant difference (*p* < 0.05) between the means of the five treatments indicated by different letters.

The root growth of sweet potato was induced significantly at 10 mg L^−1^ V treatment; however, 25, 50, and 75 mg L^−1^ V treatments presented a significant reduction in the root characteristics (Figure 2). The root length (13.1%), volume (48.6%), average diameter (13.3%), surface area (8.7%), projected area (32.5%), length per volume (15%), and crossing (17.1%) were more enhanced under 10 mg L^−1^ V than the control. The tips and forks of the roots reduced by 2.6% and 24.2%, respectively, at 10 mg L^−1^ V (Figure 2A–I). Conversely, a substantial reduction was detected from 25 to 75 mg L^−1^ V treatments, and a highest decrease in root characteristics was observed at 75 mg L^−1^ V treatment. The maximum reduction in the root length was 81%; likewise, root volume exhibited 41%, surface area 84.6%, average diameter 39.3%, projected area 80.9%, tips 82.6%, forks 89.2%, crossing 86.8%, and length per volume 81.5% reduction at 10 mg L^−1^ V treatment (Figure 2A–I).

### 3.3. Leaf Gas Exchange Elements

**Figure 3:**
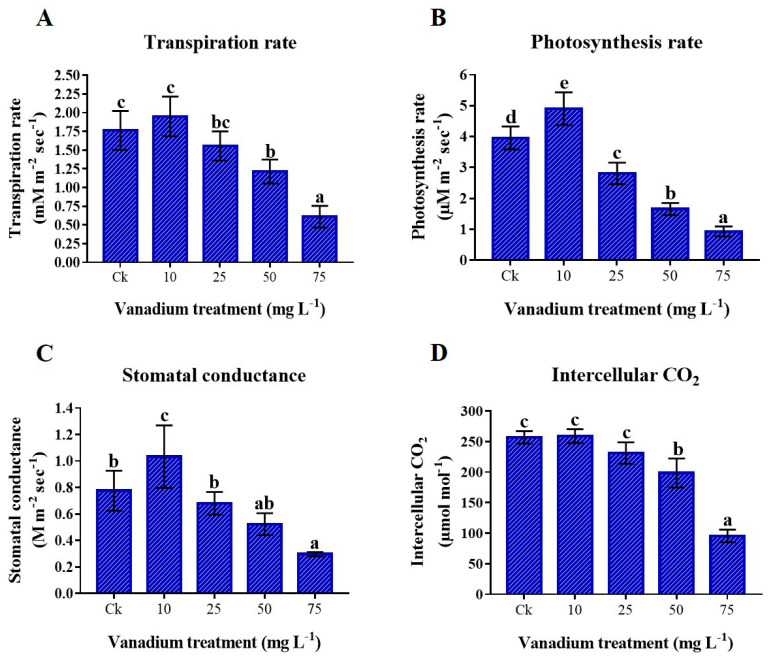
The influence of various V treatments on leaf gas exchange elements; (**A**) transpiration rate, (**B**) photosynthetic rate, (**C**) stomatal conductance, and (**D**) intercellular CO_2_. Duncan’s test indicates a significant difference (*p* < 0.05) between the means of the five treatments indicated by different alphabets.

This study showed that gas exchange elements were enhanced by the application of 10 mg L^−1^ V treatment; in contrast, a higher application of V (25, 50, and 75 mg L^−1^) showed a negative effect on the gas exchange elements of sweet potato leaves in comparison to the control plant (Figure 3). Comparing the 10 mg L^−1^ V-treated group to the control group, the transpiration rate (Tr), photosynthesis rate (Pn), stomatal conductance (Gs), and intercellular CO_2_ (Ci) increased by 10.6%, 23.9%, 33.2%, and 0.8%, respectively (Figure 3). Conversely, maximum reductions of 65.4%, 76.5%, 61.8%, and 62.8% were noticed in the Tr, Pn, Gs, and Ci, respectively when compared to the control (Figure 3).

### 3.4. Concentration, Uptake, and Translocation of Vanadium

This study depicted that V treatment significantly raised the V concentration in both shoots and roots, and maximum concentrations were recorded in 75 mg L^−1^ treated plants; 32.03 mg kg^−1^ DW in the shoots of sweet potato and 52.68 mg kg^−1^ DW in the roots of sweet potato (Table 3). Similarly, the accumulation of V was found to be higher in the roots than in the shoots. Moreover, V uptake by shoots and roots of sweet potato significantly augmented as the level of V increased. In the same way, the translocation of V from root to shoot was also raised significantly by the increment of V concentration (Table 3).

### 3.5. Photosynthetic Pigments

**Figure 4:**
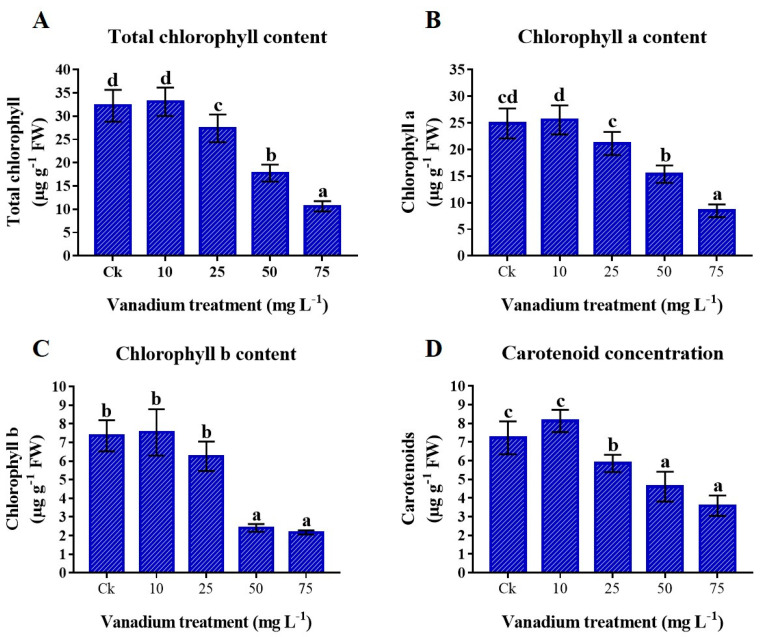
Influence of various V treatments on the photosynthetic pigments in the leaves of sweet potato. (**A**) Total chlorophyll content (T. Chl), (**B**) chlorophyll a (chl a), (**C**) chlorophyll b (chl b), and (**D**) carotenoid (Car) content. Duncan’s test indicates a significant difference (*p* < 0.05) between the means of the five treatments indicated by different alphabets.

The chlorophyll (Chl) was significantly influenced by the application of V stress. Compared to the control, adding 10 mg L^−1^ V did not significantly raise the concentration of chlorophyll and carotenoid (Car) (Figure 4). On the other hand, higher treatment of V (25, 50, and 75 mg L^−1^) showed a significant negative impact on the photosynthetic pigments, and utmost reduction was detected at 75 mg L^−1^. Compared to the control, 77% reduction in the content of total chl, 65.9% in chl a, 70.5% in chl b, and 50.3% in Car were detected (Figure 4). 

### 3.6. Lipid Peroxidation (MDA) and Reactive Oxygen Species (H_2_O_2_) Content

**Figure 5:**
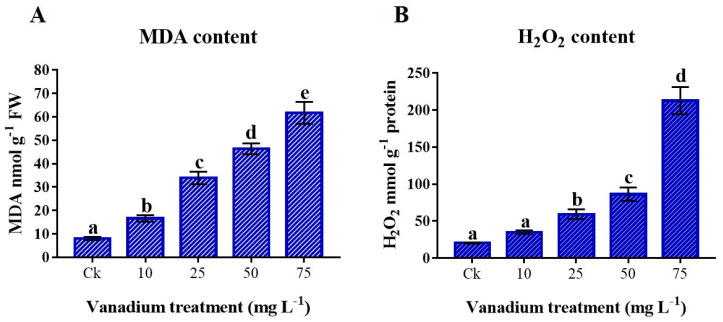
The influence of various V treatments on the production of reactive oxygen species (ROS) and lipid peroxidation in the leaves of sweet potato. (**A**) Malondialdehyde (MDA) and (**B**) hydrogen peroxide (H_2_O_2_) content. Duncan’s test specifies a significant difference (*p* < 0.05) between the means of the five treatments indicated by different alphabets.

The V treatment considerably provoked MDA and H_2_O_2_ levels in the leaves (*p* < 0.05; Figure 5A,B). The rise of V treatment caused an increase in MDA and H_2_O_2_ content; the highest MDA and H_2_O_2_ content was present in 75 mg L^−1^ V treatment compared to the control. The MDA content in 75 mg L^−1^ V was 928.9% higher than the control (Figure 5A), where the H_2_O_2_ content was 665% higher (Figure 5B).

### 3.7. Osmolytes Production

**Figure 6:**
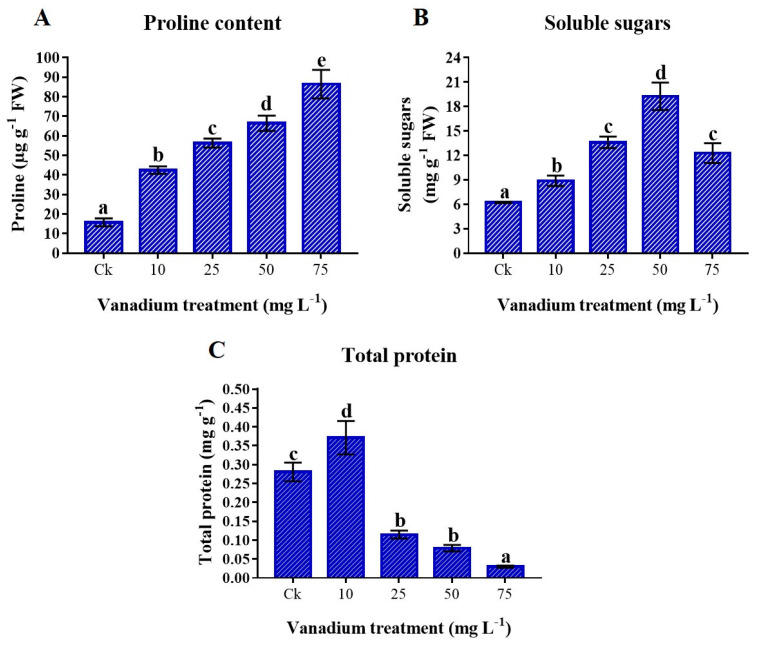
The influence of various V treatments on the osmolytes production in the leaves of sweet potato. (**A**) Proline content, (**B**) soluble sugars, and (**C**) total protein content. Duncan’s test indicates a significant difference (*p* < 0.05) between the means of the five treatments indicated by different alphabetical letters.

The proline content was significantly increased as the level of V increased (*p* < 0.05). The maximum rise of 448.3% in proline content was detected at 75 mg L^−1^ V treatment (Figure 6A). Soluble sugars were also significantly higher in V-treated plants (*p* < 0.05). The content of soluble sugars in the leaves of sweet potato was increased to 50 mg L^−1^; however, at 75 mg L^−1^ V, the content of soluble sugars decreased but was still significantly higher (97.6%) than the control plants (Figure 6B). Furthermore, the results exhibited that the total proteins were significantly increased at 10 mg L^−1^ V treatment; later, a significant decrease was observed at a higher level of V treatment (*p* < 0.05). The lowest protein content (0.029 mg g^−1^) was observed at 75 mg L^−1^ V treatment compared to the control (Figure 6C).

### 3.8. Antioxidants

**Figure 7:**
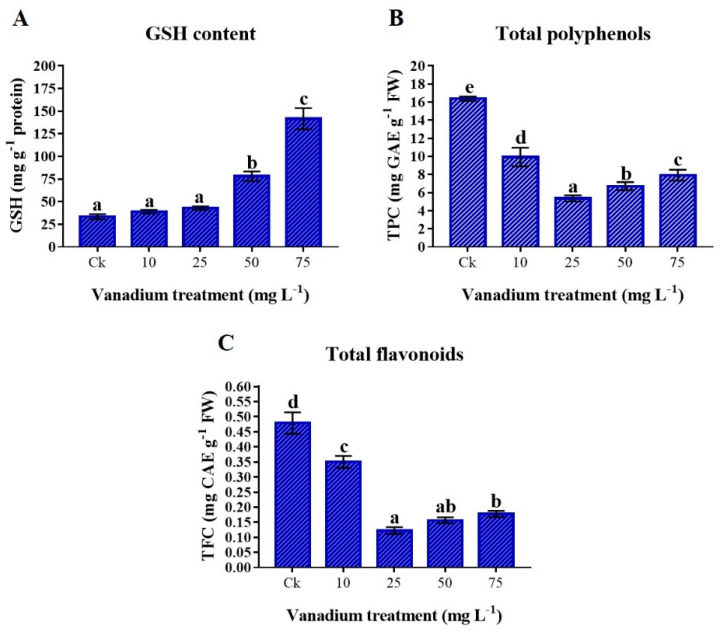
The influence of various V treatments on the antioxidants in the leaves of sweet potato. (**A**) GSH content, (**B**) total polyphenols (TPC), and (**C**) total flavonoid (TFC) content. Duncan’s test indicates a significant difference (*p* < 0.05) between the means of the five treatments indicated by different alphabetical letters.

The GSH content in the leaf of sweet potato increased with the rise of V treatment, and the maximum concentration was observed at 75 mg L^−1^ V treatment (Figure 7A). The GSH content at 10 and 25 mg L^−1^ V treatment was insignificantly increased (16.4 and 28.5%); however, a significant increase was observed at 50 and 75 mg L^−1^ V treatment, which were 134% and 324% higher than the control, respectively. Total polyphenols and flavonoid concentrations decreased significantly with the rise of V treatment (*p* < 0.05). Interestingly, total polyphenols and flavonoids drastically reduced at 10 and 25 mg L^−1^ V treatment, then again increased at 50 and 75 mg L^−1^ V treatment, however still significantly lower than in the control plants (Figure 7B,C).

### 3.9. Antioxidant Enzymes

**Figure 8:**
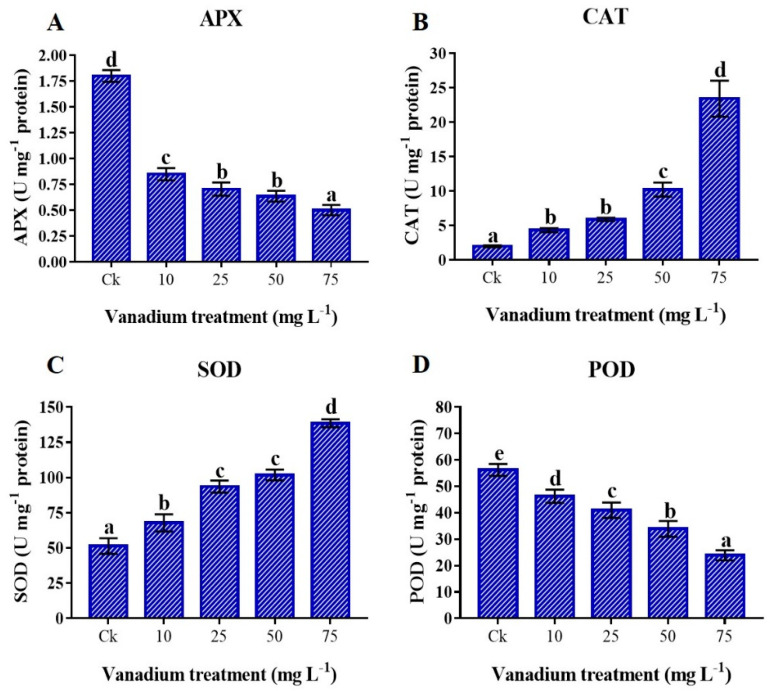
Influence of various V treatments on the antioxidant enzymes activities in the leaves of sweet potato. (**A**) APX, (**B**) CAT, (**C**) SOD, and (**D**) POD. Duncan’s test indicates a significant difference (*p* < 0.05) between the means of the five treatments indicated by different alphabetical letters.

Antioxidant enzyme activities were significantly influenced by V treatment (*p* < 0.05). We found a significant decrease in APX and POD activities with the rise of V concentration (*p* < 0.05; Figure 8A,D), and maximum reduction was detected at 75 mg L^−1^ of V treatment. As compared to the control, a 62.1% reduction in APX and a 57.5% in POD was detected at 75 mg L^−1^ V treatment. Conversely, the CAT and SOD were positively influenced by V treatment, and a significant increase was observed with the rise in V concentration (*p* < 0.05; Figure 8B,C). Furthermore, the highest activities of CAT and SOD were observed at the 75 mg L^−1^ V treatment, and a 1085% increase in CAT and a 164.7% increase in SOD were observed compared with the control.

### 3.10. Effects on Leaf Morphology

**Figure 9:**
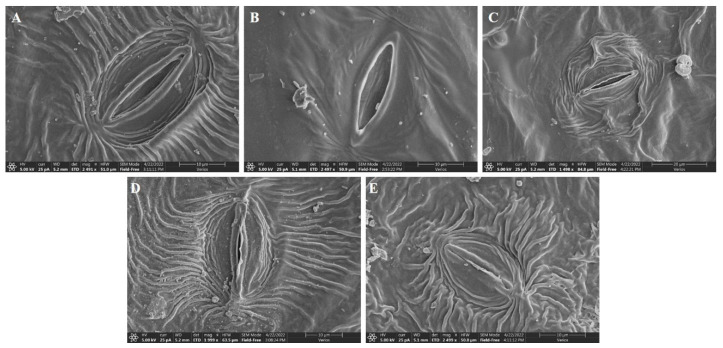
Effect of different V treatments on the stomatal traits of sweet potato leaf. (**A**) Ck, (**B**) 10 mg L^−1^, (**C**) 25 mg L^−1^, (**D**) 50 mg L^−1^, and (**E**) 75 mg L^−1^.

The present work also attempted to study the effects of V treatment on leaf morphology under SEM. The results showed that leaf morphology under 10 mg L^−1^ V treatment was statistically the same as the control plants (Figure 9). Compared to the control plants, stomatal length, width, pore length, and pore width were increased by 4.7%, 2.6%, 10.1%, and 10.1%, respectively, under 10 mg L^−1^ V treatment (Table 4). In contrast, stomata size was significantly affected under a high level of V treatment (25, 50, and 75 mg L^−1^), and the maximum deleterious effects were observed at 75 mg L^−1^ V treatment (Figure 9). Compared to the control leaf, stomata length under 75 mg L^−1^ V treatment was reduced by 47.2%; likewise, the width of the stomata was decreased by 80.1%, pore length by 71%, and pore width by 87.1%. Closed and small stomata were observed due to stress conditions, which showed small stomatal openings. The result indicated that V induced stomatal closing and reduced its size.

### 3.11. Pearson’s Correlation and Heat-map Analysis

**Figure 10:**
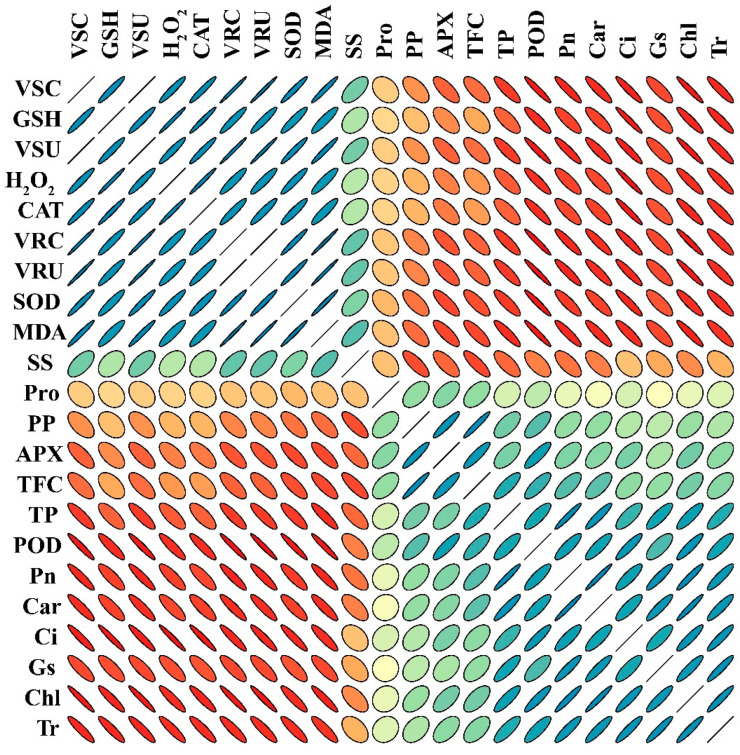
Pearson’s correlation analysis (PCA) (*p* < 0.05) was calculated among different traits of V-treated sweet potato seedling. VSC (V shoot concentration), GSH (reduced glutathione), VSU (V shoot uptake), H_2_O_2_ (hydrogen peroxide), CAT (catalase), VRC (V root concentration), VRU (V root uptake in root), SOD (superoxide dismutase), MDA (malondialdehyde), SS (soluble sugars), Pro (proline), PP (total polyphenols), APX (ascorbate peroxidase), TF (total flavonoids), TP (total proteins), POD (peroxidase), Pn (photosynthetic assimilation), Car (carotenoids), Ci (Intercellular CO_2_), Gs (stomatal conductance), Chl (total chlorophyll), and Tr (transpiration rate). Measured determinants and strength correlation matrix. The color code for Pearson correlation coefficients (r), with r = 2, r = 0, and r = −2 denoting red, orange, green, and gray, respectively. A stronger association is denoted by better anisotropy, and the slope of the corresponding line or ellipse represents the trend of that association (positive or negative). Additionally, the ellipses’ direction and anisotropy indicate the slope and strength of the relationship. According to the first principal component order, variables were arranged.

The negative correlation between physiological parameters and osmolytes, GSH, CAT, and SOD in sweet potato plants treated with different concentrations of V demonstrates the significant reduction in the plant’s phenotypic and physiological traits (Figure 10). All plant phenotypic parameters and root and shoot concentrations of V were negatively correlated, showing that V treatment adversely affected plant growth and development. Similarly, osmolytes, GSH, CAT, SOD, and V concentrations and uptake in sweet potato showed a negative correlation with photosynthetic pigments, assimilation, and stomatal traits (Figure 10). However, the phenotypic parameters showed a positive correlation with photosynthetic pigments and assimilation, indicating that plants can grow larger and produce more biomass at a high rate of photosynthetic pigments.

**Figure 11:**
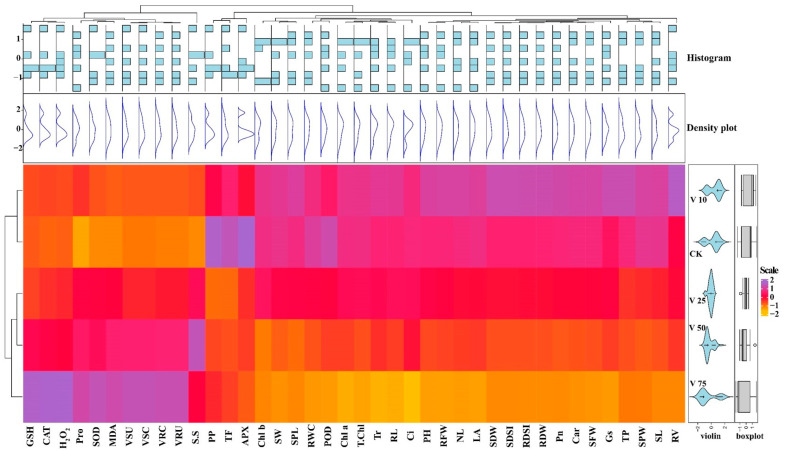
Heatmap-histogram correlation between studied physiological and biochemical parameters under various V treatments in sweet potato.

A heatmap-histogram analysis of different growth traits of sweet potato under different levels of V treatment was also constructed (Figure 11). A significant difference was observed with different colors in the different V treatments and responses of different physiological and biochemical parameters, as well as V concentration and uptake. However, traits with red color indicate insignificant differences within the V treatments. This heatmap-histogram showed a noticeable difference among the growth traits and uptake of V in the sweet potato plant.

## 4. Discussion

Globally, agricultural soil has been polluted with several kinds of soil pollutants with anthropogenic activities. Several studies have indicated that heavy metals, such as Cd, Ni, Pb, and V, are the primary cause of soil pollution. V used in the steel industries and accumulation in the agricultural land and water gained consideration by researchers in recent years [35,36,37]. V accumulation showed a deleterious effect on living organisms, including plants, animals, and humans. In order to better understand the mechanisms underlying V toxicity in sweet potato, we investigated the phenotypic, physiological, and biochemical processes under various levels of V treatments. Plant growth and biomass are not only used to study different types of heavy metal stress, but also used to evaluate the tolerance level of plants against them. Heavy metals cause plant toxicity and negatively affect the plant length and fresh and dry biomass [38]. However, its toxicity varies with the plant species, chemical structure and formula, concentration, and recurrence of use [39]. However, at higher concentrations, V severely inhibits plant growth and development [40]. In the present study, we observed an improvement in the growth of sweet potato under 10 mg L^−1^ V treatment compared to the control plant (Figure 1). According to some studies, plants treated with V had increased height, growth, and fresh and dry biomass [11,12]. Aihemaiti et al. stated that plants generate more biomass at low levels of V due to enhanced chlorophyll biosynthesis [22]. However, many studies reported a decrease in the plant height, growth, and fresh biomass under a higher level of V stress [38,41], and the impact varies from organ to organ of the plants [8]. Similarly, the current study depicted that 25, 50, and 75 mg L^−1^ V treatment considerably reduced the growth of sweet potato, and maximum reduction was observed at 75 mg L^−1^ (Table 1 and Table 2). The SDSI and RDSI increased at 10 mg L^−1^; in contrast, a negative correlation was observed with a further increase of the V level (25, 50, and 75 mg L^−1^) in the growth medium. A higher level of V stress initiated an ionic imbalance and interrupted their function in metabolic pathways, which eventually affected the process of growth and development of the plant. RWC is a simple and reliable parameter for calculating plant stress. Under various V treatments, we found a decrease in the RWC, indicating that the sweet potato plants were under stress. Osmotic adjustment is impaired in sensitive plant species. A previous study on lettuce also showed a decrease in water content with an increase of V concentration [42]. Under heavy metal stress, various studies also reported a reduction in RWC in the leaves of barley and maize [43,44]. The present study showed that a low level of V treatment (10 mg L^−1^) induced the root traits of sweet potato (Figure 2). Variations in root size and morphology can improve ionic uptake and translocation, ultimately enhancing plant growth and development [45]. A previous study also reported the expansion of root traits under low V treatment (10 mg L^−1^) [11]. In contrast, a higher level of V (25, 50, and 75 mg L^−1^) caused a decrease in these root traits (Figure 2). At a higher level of V treatment, the plant produced comparatively shorter, less lateral, and coralloid structural roots. The decline in root traits under high V levels might have been due to the disruption in mitotic cell division, possibly hampering root tip development [46,47]. Previous studies also reported the decrease of root traits under a higher level of V treatment in watermelon, pepper, and rice [11,13,20]. In addition, V application significantly reduced root morphological traits in many plants [22]; these studies are in agreement with the findings of the present study. 

Photosynthetic assimilation is the plant’s ability to use CO_2_ and perform many metabolic activities [48]. Heavy metal stress drastically affects photosynthesis and significantly affects carbohydrate synthesis. In the current study, we found an increase in these gas exchange elements under 10 mg L^−1^ treatment; however, a significant decrease in these gas exchange elements was observed at a higher level of V stress (Figure 3). A previous study reported a minor increase in the photosynthetic rate and intercellular CO_2_ at a low level of V (10 mg L^−1^) treatment; however, a higher level of V showed a significant reduction in these gas exchange elements in pepper plants [11]. Similarly, another study revealed that V treatment in rice plants caused a reduction in gas exchange elements, which agrees with the current study [13]. At high concentrations, V imposes adverse effects on the physiological processes and photosynthetic assimilation, limiting energy production, and impairing biomass and development of plants [49,50]. V stress possibly reduces photosynthetic activities by affecting the chloroplast and ultrastructures and also disturbs their electron transport mechanisms. 

The leaf stomata are responsible for regulating transpiration and CO_2_ transport under adverse environmental conditions [51]. The plant experiences cytotoxicity due to the increased concentration of heavy metals, which affects ionic absorption, cell cycle arrest, lipid peroxidation, and ultimately results in cell death [52,53]. Heavy metal interaction with guard cells triggers stomatal closure [54]. V toxicity mediates stomatal abnormalities, such as a reduction in the stomatal length, width, pore length, and width of guard cells (Figure 9 and Table 4). A study reported that the reduction in the stomata size is linked with more distorted stomata under an elevated level of heavy metals in the leaves [30,55]. Photosynthesis, transpiration, and gas exchange can be adversely affected by reductions in stomatal size and closure frequency. From the present study, it has been elucidated that increasing V level decreases stomatal size and closing stomata, which reflects a decrease in photosynthetic assimilation, transpiration rate, and gas exchange. 

In the present study, the V concentration, uptake, and translocation had a positive correlation with the rise of V application, and the roots showed higher V content than the shoots (Table 3). The concentration, uptake, and translocation of V to the aerial part are reduced by chelation and fixation of V with a polar compound, stimulation of calcium with a stable compound, and root and stem compartmentalization [56]. A previous study reported that tomato plants treated with V have more V in the roots than in the leaves [36]. In the same way, lettuce, tobacco, alfalfa, milkvetch root, and swamp morning glory treated with V also showed the same trend, and they have more V accumulation in roots than the leaves [1,10,35,42]. Generally, the increase in chlorophyll level characterizes plant photosynthesis assimilation and growth. The reduction in the chlorophyll content was found under different environmental stresses. A higher level of V treatment (25, 50, and 75 mg L^−1^) reduced the T.Chl, Chl a, b, and carotenoid content (Figure 4). Similarly, previous studies revealed a significant reduction in the chlorophyll content under exposure to V stress in watermelon and pepper [11,13,20]. The reduction in photosynthetic pigments under V stress might be due to the variations in the membrane permeability and the devastation of the elements prompted by oxidative stress [57]. This increase in ROS production causes a reduction in chlorophyll content [15].

Lipids peroxidation is an indication of oxidative stress that is induced by different abiotic stress, and the higher MDA level also indicates cell membrane injury; it is a well-established sign for assessing plants introduced to metal stress [32]. The current study described that the V application considerably boosted the MDA content of sweet potato (Figure 5A). Similarly, previous studies described that V-treated rice and pepper seedlings exhibited an increase in the MDA content [11,13]. Plants produce ROS due to the reaction of heavy metals and fatty acids [58]. H_2_O_2_ is the main component of ROS and its production increases with heavy metal stress. The current study also reported that an increase in V stress in sweet potato causes a significant increment in H_2_O_2_ level (Figure 5B). Previous studies also observed a significant increment in the H_2_O_2_ level when exposed to V stress in rice, tomato, and watermelon plants [13,20,36]. A review article by Chen et al. also highlighted that many articles followed the same pattern of increasing MDA and H_2_O_2_ under V stress [59]. To deal with these circumstances, the plant possesses a defense mechanism, including osmolytes, antioxidants, and enzymes.

The reduction of osmolytes, such as proline, soluble sugars, and proteins are linked with the leaf water content, which could lead to cellular desiccation and osmotic stress in sweet potato (Figure 6). According to the previous literature, plants exhibit a similar pattern by increasing proline and soluble sugars in response to increased cellular desiccation under V stress [6,11,22,42]. These osmolytes increase under abiotic stress conditions [17,32]. Moreover, these osmolytes might not only assist in protecting plant cellular membranes, but also help in maintaining turgor pressure, which minimizes the deleterious effect of vanadium toxicity. The total proteins are sensitive to heavy metal stress, and different studies reported a reduction of proteins with increased metal stress [13]. The present study showed a significant decrease in total protein content at a higher level of V treatment (25, 50, and 75 mg L^−1^) (Figure 7A). Heavy metal stress reduces the proteins by prompting toxic effects, damaging the ability of many enzymes with functional thiol groups [60]. The results of the present study agree with the reports of the Refs. [36,61], who stated that total protein content was reduced under V stress. The GSH can enhance the tolerance of plants under metal stress. Furthermore, GSH acts as a ROS scavenger, detoxifying the O_2_^•−^ and HO^•^ radicals [32]. This study depicted an increase of GSH under V stress (Figure 7B). GSH levels increase under heavy metal stress due to increased activities of γ-glutamylcysteine synthetase and glutathione synthetase [62]. Our findings agree with previous reports, which described the increment in GSH content under heavy metal stress [61,63]. The secondary metabolites, such as polyphenols and flavonoids, not only enhance the enzymatic activities of the plants but also play a vital role as antioxidants in stress environments [17]. Izbiańska et al. and Kisa et al. reported a decrease in phenolics under heavy metal stress [64,65]. This study depicted that V-treated plants have a significant reduced level of polyphenols and flavonoids (Figure 7C,D). This reduction in the polyphenols and flavonoids biosynthesis under V stress could be due to the decreased activity of essential enzymes of phenylpropanoid pathways [65,66]. Antioxidant enzymes are essential in reducing ROS production and oxidative stress under different environmental stress conditions [17,32]. In this study, we found a decrease in the activity of POD and APX with the rise in V level. In contrast, CAT and SOD significantly increased with the increase of V treatment (Figure 8). This increase in antioxidant enzyme activity may be attributed to the effect of the V ion on free-oxygen radical production. Tobacco plants treated with V have depicted an increment of CAT and SOD activities [10]. Likewise, watermelon and pepper plants under V stress have also depicted increased CAT and SOD activities [11,20]. The results of the current study also agree with studies on rice, chickpeas, oilseed, and tomato under V, Se, and Ni stress, respectively. [41,61,67,68]. Similarly, many other plants have also shown increased activities of these antioxidant enzymes under different levels of V treatments [59]. The antioxidant enzyme activities are powerful ROS scavengers and regulate the plant’s cellular membranes under abiotic stress conditions.

## 5. Conclusions

In the present study, we found that 10 mg L^−1^ V treatment improved the growth and biomass of the sweet potato plant. However, V at higher levels (25, 50, and 75 mg L^−1^) significantly reduced the growth of sweet potato by altering its physiological and biochemical mechanisms. The higher level of V treatments (25, 50, and 75 mg L^−1^) significantly reduced the RWC, chlorophyll content, gas exchange elements of leaf, and stomatal traits, which eventually affected the growth of sweet potato. The decline in root morphological traits was observed at the higher V treatment, and we also detected maximum V concentration and uptake in the roots than in the shoots of sweet potato. It was also observed that the V application increased ROS production. Overall, this study will help to understand the physiological tolerance mechanism of V in sweet potato plants. In this study, we focused on the effect of V stress on the morphological and physiological aspects of sweet potato. Besides, this study was conducted in a hydroponically controlled environment, and thus, open-field experiments are encouraged to unravel the more precise effects of V on sweet potato, as in open-field conditions the plants are exposed to several other biotic and abiotic stresses. Moreover, further studies are also required to investigate the molecular mechanism underlying cell death induced by V toxicity in the sweet potato plant. Furthermore, we suggest using various chemicals, phytohormones, and nanoparticles to prevent V toxicity and stimulate sweet potato growth and production.

## Figures and Tables

**Table 1:** The influence of various V treatments on growth parameters of sweet potato.

Vanadium (mg L^−1^)	Height (cm)	Leaf Area (cm^2^)	Number of Leaf	Shoot FW (g)	Root FW (g)	RWC (%)
Ck	42.67 ± 4.0 ^cd^	69.55 ± 8.0 ^c^	5.67 ± 0.6 ^c^	5.18 ± 0.4 ^d^	2.55 ± 0.5 ^cd^	92.98 ± 0.88 ^e^
10	46.33 ± 3.1 ^d^	74.86 ± 5.8 ^c^	6.33 ± 0.6 ^c^	5.90 ± 0.5 ^e^	2.97 ± 0.4 ^d^	90.18 ± 1.66 ^d^
25	38.00 ± 4.6 ^bc^	52.27 ± 4.3 ^b^	4.33 ± 0.6 ^b^	4.09 ± 0.4 ^c^	2.03 ± 0.3 ^bc^	83.89 ± 0.68 ^c^
50	31.67 ± 3.5 ^ab^	44.24 ± 5.6 ^b^	3.67 ± 0.6 ^b^	3.08 ± 0.3 ^b^	1.49 ± 0.2 ^b^	76.52 ± 1.33 ^b^
75	25.33 ± 4.2 ^a^	27.03 ± 4.7 ^a^	2.33 ± 0.6 ^a^	2.19 ± 0.2 ^a^	0.80 ± 0.1 ^a^	70.97 ± 1.12 ^a^

Duncan’s test indicates a significant difference (*p* < 0.05) between the means of the five treatments indicated by different alphabetical letters.

**Table 2:** The influence of various V treatments on root and shoot dry weight and root and shoot dry weight susceptibility index of sweet potato.

Vanadium (mg L^−1^)	Shoot DW (g)	Root DW (g)	Root-Shoot Ratio	SDSI	RDSI
Ck	0.518 ± 0.05 ^d^	0.324 ± 0.07 ^d^	0.623 ± 0.10 ^bc^	100	100
10	0.651 ± 0.09 ^e^	0.418 ± 0.05 ^e^	0.643 ± 0.01 ^c^	125.491 ± 12.3 ^d^	130.297 ± 10.5 ^d^
25	0.387 ± 0.03 ^c^	0.225 ±0.02 ^c^	0.582 ± 0.01 ^bc^	74.819 ± 5.5 ^c^	70.663 ± 8.8 ^c^
50	0.288 ± 0.02 ^b^	0.148 ± 0.03 ^b^	0.511 ± 0.05 ^b^	55.725 ± 4.6 ^b^	45.967 ± 3.6 ^b^
75	0.196 ± 0.02 ^a^	0.074 ± 0.01 ^a^	0.382 ± 0.07 ^a^	37.881 ± 2.8 ^a^	23.727 ± 6.7 ^a^

Duncan’s test indicates a significant difference (*p* < 0.05) between the means of the five treatments indicated by different alphabetical letters.

**Table 3:** The influence of various V treatments on the concentrations, uptake, and translocation (root to shoot) of V.

Vanadium (mg L^−1^)	Concentration (mg kg^−1^ DW)		Uptake (mg kg^−1^ DW)		Translocation (Root to Shoot)
	Shoot	Root	Shoot	Root	
Ck	0.07 ± 0.01 ^a^	0.89 ± 0.30 ^a^	0.01 ± 0.00 ^a^	0.09 ± 0.02 ^a^	0.08 ± 0.01 ^a^
10	3.53 ± 0.30 ^b^	7.91 ± 0.72 ^b^	0.37 ± 0.03 ^b^	0.82 ± 0.06 ^b^	0.45 ± 0.00 ^b^
25	9.50 ± 0.79 ^c^	19.19 ± 1.62 ^c^	0.99 ± 0.09 ^c^	1.99 ± 0.19 ^c^	0.50 ± 0.01 ^bc^
50	21.22 ± 1.49 ^d^	37.41 ± 3.02 ^d^	2.20 ± 0.17 ^d^	3.88 ± 0.26 ^d^	0.57 ± 0.08 ^cd^
75	32.03 ± 2.41 ^e^	52.68 ± 3.27 ^e^	3.32 ± 0.27 ^e^	5.46 ± 0.39 ^e^	0.61 ± 0.08 ^d^

Duncan’s test indicates a significant difference (*p* < 0.05) between the means of the five treatments indicated by different alphabets.

**Table 4:** Effect of different V treatments on the stomatal traits of sweet potato leaf.

Vanadium (mg L^−1^)	Stomata Length (µm)	Stomata Width (µm)	Stomatal Pore Length (µm)	Stomatal Pore Width (µm)
Ck	25.52 ± 2.45 ^c^	15.52 ± 1.92 ^c^	16.30 ± 2.11 ^c^	3.92 ± 1.08 ^b^
10	26.71 ± 2.97 ^c^	15.93 ± 1.78 ^c^	18.16 ± 1.31 ^c^	4.32 ± 0.92 ^b^
25	18.62 ± 2.31 ^b^	11.04 ± 1.51 ^b^	12.26 ± 1.63 ^b^	1.69 ± 0.47 ^a^
50	16.22 ± 1.73 ^ab^	5.70 ± 1.01 ^a^	6.19 ± 1.49 ^a^	0.95 ± 0.36 ^a^
75	13.47 ± 1.69 ^a^	3.08 ± 0.90 ^a^	4.72 ± 1.01 ^a^	0.51 ± 0.16 ^a^

Duncan’s test specifies a significant difference (*p* < 0.05) between the means of the five treatments indicated by different alphabets.

## Data Availability

The data presented in this article will be made available without any reservation.

## References

[1] Gan C., Chen T., Yang J. (2021). Growth responses and accumulation of vanadium in alfalfa, milkvetch root, and swamp morning glory and their potential in phytoremediation. Bull. Environ. Contam. Toxicol..

[2] Amorim F.A.C., Welz B., Costa A.C.S., Lepri F.G., Vale M.G.R., Ferreira S.L.C. (2007). Determination of vanadium in petroleum and petroleum products using atomic spectrometric techniques. Talanta.

[3] Yang J., Teng Y., Wu J., Chen H., Wang G., Song L., Yue W., Zuo R., Zhai Y. (2017). Current status and associated human health risk of vanadium in soil in China. Chemosphere.

[4] Chen L., Zhu Y., Luo H., Yang J. (2020). Characteristic of adsorption, desorption, and co-transport of vanadium on humic acid colloid. Ecotoxicol. Environ. Saf..

[5] Tian L.-Y., Yang J.-Y., Huang J.-H. (2015). Uptake and speciation of vanadium in the rhizosphere soils of rape (*Brassica juncea* L.). Environ. Sci. Pollut. Res..

[6] Wu Z., Zhang Y., Yang J., Zhou Y., Wang C. (2021). Effect of vanadium on testa, seed germination, and subsequent seedling growth of alfalfa (*Medicago sativa* L.). J. Plant Growth Regul..

[7] He W., Liao W., Yang J., Jeyakumar P., Anderson C. (2020). Removal of vanadium from aquatic environment using phosphoric acid modified rice straw. Bioremediat. J..

[8] Imtiaz M., Rizwan M.S., Xiong S., Li H., Ashraf M., Shahzad S.M., Shahzad M., Rizwan M., Tu S. (2015). Vanadium, recent advancements and research prospects: A review. Environ. Int..

[9] Larsson M.A., Baken S., Gustafsson J.P., Hadialhejazi G., Smolders E. (2013). Vanadium bioavailability and toxicity to soil microorganisms and plants. Environ. Toxicol. Chem..

[10] Wu Z., Yang J., Zhang Y., Wang C., Guo S., Yu Y. (2021). Growth responses, accumulation, translocation and distribution of vanadium in tobacco and its potential in phytoremediation. Ecotoxicol. Environ. Saf..

[11] Altaf M.A., Shu H., Hao Y., Zhou Y., Mumtaz M.A., Wang Z. (2021). Vanadium toxicity induced changes in growth, antioxidant profiling, and vanadium uptake in pepper (*Capsicum annum* L.) seedlings. Horticulturae.

[12] Chen T., Li T.-Q., Yang J.-Y. (2016). Damage suffered by swamp morning glory (*Ipomoea aquatica* Forsk) exposed to vanadium (V). Environ. Toxicol. Chem..

[13] Altaf M.M., Diao X.P., Ur Rehman A., Imtiaz M., Shakoor A., Altaf M.A., Younis H., Fu P., Ghani M.U. (2020). Effect of vanadium on growth, photosynthesis, reactive oxygen species, antioxidant enzymes, and cell death of rice. J. Soil Sci. Plant Nutr..

[14] Reiter R., Tan D.-X., Zhou Z., Cruz M., Fuentes-Broto L., Galano A. (2015). Phytomelatonin: Assisting plants to survive and thrive. Molecules.

[15] Sridhara Chary N., Kamala C.T., Samuel Suman Raj D. (2008). Assessing risk of heavy metals from consuming food grown on sewage irrigated soils and food chain transfer. Ecotoxicol. Environ. Saf..

[16] Kumar S., Li G., Huang X., Ji Q., Zhou K., Hou H., Ke W., Yang J. (2021). Phenotypic, nutritional, and antioxidant characterization of blanched *Oenanthe javanica* for preferable cultivar. Front. Plant Sci..

[17] Kumar S., Li G., Yang J., Huang X., Ji Q., Zhou K., Khan S., Ke W., Hou H. (2020). Investigation of an antioxidative system for salinity tolerance in *Oenanthe javanica*. Antioxidants.

[18] Imtiaz M., Mushtaq M.A., Rizwan M.S., Arif M.S., Yousaf B., Ashraf M., Shuanglian X., Rizwan M., Mehmood S., Tu S. (2016). Comparison of antioxidant enzyme activities and DNA damage in chickpea (*Cicer arietinum* L.) genotypes exposed to vanadium. Environ. Sci. Pollut. Res..

[19] Altaf M.A., Shahid R., Ren M.-X., Altaf M.M., Jahan M.S., Khan L.U. (2021). Melatonin mitigates nickel toxicity by improving nutrient uptake fluxes, root architecture system, photosynthesis, and antioxidant potential in tomato seedling. J. Soil Sci. Plant Nutr..

[20] Nawaz M.A., Jiao Y., Chen C., Shireen F., Zheng Z., Imtiaz M., Bie Z., Huang Y. (2018). Melatonin pretreatment improves vanadium stress tolerance of watermelon seedlings by reducing vanadium concentration in the leaves and regulating melatonin biosynthesis and antioxidant-related gene expression. J. Plant Physiol..

[21] Andresen E., Küpper H., Sigel A., Sigel H., Sigel R.K.O. (2013). Cadmium Toxicity in Plants. Cadmium: From Toxicity to Essentiality.

[22] Aihemaiti A., Gao Y., Meng Y., Chen X., Liu J., Xiang H., Xu Y., Jiang J. (2020). Review of plant-vanadium physiological interactions, bioaccumulation, and bioremediation of vanadium-contaminated sites. Sci. Total Environ..

[23] Kumar S., Wang M., Liu Y., Fahad S., Qayyum A., Jadoon S.A., Chen Y., Zhu G. (2022). Nickel toxicity alters growth patterns and induces oxidative stress response in sweetpotato. Front. Plant Sci..

[24] Laurie S.M., Faber M., Claasen N. (2018). Incorporating orange-fleshed sweet potato into the food system as a strategy for improved nutrition: The context of South Africa. Food Res. Int..

[25] Mussoline W.A., Bohac J.R., Boman B.J., Trupia S., Wilkie A.C. (2017). Agronomic productivity, bioethanol potential and postharvest storability of an industrial sweetpotato cultivar. Ind. Crops Prod..

[26] Shekhar S., Mishra D., Buragohain A.K., Chakraborty S., Chakraborty N. (2015). Comparative analysis of phytochemicals and nutrient availability in two contrasting cultivars of sweet potato (*Ipomoea batatas* L.). Food Chem..

[27] Fu Z.F., Tu Z.C., Zhang L., Wang H., Wen Q.H., Huang T. (2016). Antioxidant activities and polyphenols of sweet potato (*Ipomoea batatas* L.) leaves extracted with solvents of various polarities. Food Biosci..

[28] Sun H., Mu T., Xi L., Zhang M., Chen J. (2014). Sweet potato (*Ipomoea batatas* L.) leaves as nutritional and functional foods. Food Chem..

[29] Kurata R., Adachi M., Yamakawa O., Yoshimoto M. (2007). Growth suppression of human cancer cells by polyphenolics from sweetpotato (*Ipomoea batatas* L.) leaves. J. Agric. Food Chem..

[30] Kumar S., Wang M., Fahad S., Qayyum A., Chen Y., Zhu G. (2022). Chromium induces toxicity at different phenotypic, physiological, biochemical, and ultrastructural levels in Sweet potato (*Ipomoea batatas* L.) plants. Int. J. Mol. Sci..

[31] García-Jiménez A., Trejo-Téllez L.I., Guillén-Sánchez D., Gómez-Merino F.C. (2018). Vanadium stimulates pepper plant growth and flowering, increases concentrations of amino acids, sugars and chlorophylls, and modifies nutrient concentrations. PLoS ONE.

[32] Kumar S., Li G., Yang J., Huang X., Ji Q., Liu Z., Ke W., Hou H. (2021). Effect of salt stress on growth, physiological parameters, and ionic concentration of water dropwort (*Oenanthe javanica*) cultivars. Front. Plant Sci..

[33] Yang J., Li G., Bishopp A., Heenatigala P.P.M., Hu S., Chen Y., Wu Z., Kumar S., Duan P., Yao L. (2018). A comparison of growth on mercuric chloride for three *Lemnaceae* species reveals differences in growth dynamics that effect their suitability for use in either monitoring or remediating ecosystems contaminated with mercury. Front. Chem..

[34] Kumar S., Huang X., Ji Q., Qayyum A., Zhou K., Ke W., Zhu H., Zhu G. (2022). Influence of blanching on the gene expression profile of phenylpropanoid, flavonoid and vitamin biosynthesis, and their accumulation in *Oenanthe javanica*. Antioxidants.

[35] Gan C., Chen T., Yang J. (2020). Remediation of vanadium contaminated soil by alfalfa (*Medicago sativa* L.) combined with vanadium-resistant bacterial strain. Environ. Technol. Innov..

[36] Altaf M.A., Shahid R., Ren M.-X., Khan L.U., Altaf M.M., Jahan M.S., Nawaz M.A., Naz S., Shahid S., Lal M.K. (2021). Protective mechanisms of melatonin against vanadium phytotoxicity in tomato seedlings: Insights into nutritional status, photosynthesis, root architecture system, and antioxidant machinery. J. Plant Growth Regul..

[37] Yu Y., Li J., Liao Y., Yang J. (2020). Effectiveness, stabilization, and potential feasible analysis of a biochar material on simultaneous remediation and quality improvement of vanadium contaminated soil. J. Clean. Prod..

[38] Xuebin Q., Yatao X., Ahmad M.I., Shehzad M., Zain M. (2020). Silicon and its application methods improve physiological traits and antioxidants in *Triticum aestivum* (L.) under cadmium stress. J. Soil Sci. Plant Nutr..

[39] Pilon-Smits E.A., Quinn C.F., Tapken W., Malagoli M., Schiavon M. (2009). Physiological functions of beneficial elements. Curr. Opin. Plant Biol..

[40] Saldaña-Sánchez W.D., León-Morales J.M., López-Bibiano Y., Hernández-Hernández M., Langarica-Velázquez E.C., García-Morales S. (2019). Effect of V, Se, and Ce on growth, photosynthetic pigments, and total phenol content of tomato and pepper seedlings. J. Soil Sci. Plant Nutr..

[41] Yuan Y., Imtiaz M., Rizwan M., Dong X., Tu S. (2020). Effect of vanadium on germination, growth and activities of amylase and antioxidant enzymes in genotypes of rice. Int. J. Environ. Sci. Technol..

[42] Wu Z., Zhang Y., Yang J., Jia Z. (2022). Effect of vanadium on *Lactuca sativa* L. growth and associated health risk for human due to consumption of the vegetable. Environ. Sci. Pollut. Res..

[43] González A., Gil-Díaz M., Lobo M.C. (2015). Response of two barley cultivars to increasing concentrations of cadmium or chromium in soil during the growing period. Biol. Trace Elem. Res..

[44] Bashir M.A., Wang X., Naveed M., Mustafa A., Ashraf S., Samreen T., Nadeem S.M., Jamil M. (2021). Biochar mediated-alleviation of chromium stress and growth improvement of different maize cultivars in tannery polluted soils. Int. J. Environ. Res. Public Health.

[45] Wissuwa M., Kretzschmar T., Rose T.J. (2016). From promise to application: Root traits for enhanced nutrient capture in rice breeding. J. Exp. Bot..

[46] Meisch H.-U., Benzschawel H., Bielig H.-J. (1977). The role of vanadium in green plants. Arch. Microbiol..

[47] Yang J., Wang M., Jia Y., Gou M., Zeyer J. (2017). Toxicity of vanadium in soil on soybean at different growth stages. Environ. Pollut..

[48] Guo Y., Liu Y., Wang R., Wang S., Lu X., Wang B. (2015). Effect of mercury stress on photosynthetic characteristics of two kinds of warm season turf grass. Int. J. Environ. Monit. Anal..

[49] Abedini M., Mohammadian F. (2018). Vanadium effects on phenolic content and photosynthetic pigments of sunflower. South-West. J. Hortic. Biol. Environ..

[50] Yang J.Y., Tang Y. (2015). Accumulation and biotransformation of vanadium in *Opuntia microdasys*. Bull. Environ. Contam. Toxicol..

[51] Khan M.N., Zhang J., Luo T., Liu J., Rizwan M., Fahad S., Xu Z., Hu L. (2019). Seed priming with melatonin coping drought stress in rapeseed by regulating reactive oxygen species detoxification: Antioxidant defense system, osmotic adjustment, stomatal traits and chloroplast ultrastructure perseveration. Ind. Crops Prod..

[52] Balasaraswathi K., Jayaveni S., Sridevi J., Sujatha D., Phebe Aaron K., Rose C. (2017). Cr–induced cellular injury and necrosis in *Glycine max* L.: Biochemical mechanism of oxidative damage in chloroplast. Plant Physiol. Biochem..

[53] Wakeel A., Xu M., Gan Y. (2020). Chromium-induced reactive oxygen species accumulation by altering the enzymatic antioxidant system and associated cytotoxic, genotoxic, ultrastructural, and photosynthetic changes in plants. Int. J. Mol. Sci..

[54] Rucińska-Sobkowiak R. (2016). Water relations in plants subjected to heavy metal stresses. Acta Physiol. Plant..

[55] Gautam V., Kohli S.K., Kapoor D., Bakshi P., Sharma P., Arora S., Bhardwaj R., Ahmad P. (2021). Stress protective effect of *Rhododendron arboreum* leaves (MEL) on chromium-treated *Vigna radiata* plants. J. Plant Growth Regul..

[56] Kaplan D.I., Adriano D.C., Carlson C.L., Sajwan K.S. (1990). Vanadium: Toxicity and accumulation by beans. Water. Air. Soil Pollut..

[57] Aihemaiti A., Jiang J., Blaney L., Zou Q., Gao Y., Meng Y., Yang M., Xu Y. (2019). The detoxification effect of liquid digestate on vanadium toxicity to seed germination and seedling growth of dog’s tail grass. J. Hazard. Mater..

[58] Shah K., Kumar R.G., Verma S., Dubey R. (2001). Effect of cadmium on lipid peroxidation, superoxide anion generation and activities of antioxidant enzymes in growing rice seedlings. Plant Sci..

[59] Chen L., Liu J., Hu W., Gao J., Yang J. (2021). Vanadium in soil-plant system: Source, fate, toxicity, and bioremediation. J. Hazard. Mater..

[60] Tanyolaç D., Ekmekçi Y., Ünalan Ş. (2007). Changes in photochemical and antioxidant enzyme activities in maize (*Zea mays* L.) leaves exposed to excess copper. Chemosphere.

[61] Imtiaz M., Tu S., Xie Z., Han D., Ashraf M., Rizwan M.S. (2015). Growth, V uptake, and antioxidant enzymes responses of chickpea (*Cicer arietinum* L.) genotypes under vanadium stress. Plant Soil.

[62] Mendoza-Cózatl D.G., Moreno-Sánchez R. (2006). Control of glutathione and phytochelatin synthesis under cadmium stress. Pathway modeling for plants. J. Theor. Biol..

[63] Zeng F., Qiu B., Wu X., Niu S., Wu F., Zhang G. (2012). Glutathione-mediated alleviation of chromium toxicity in rice plants. Biol. Trace Elem. Res..

[64] Izbiańska K., Arasimowicz-Jelonek M., Deckert J. (2014). Phenylpropanoid pathway metabolites promote tolerance response of lupine roots to lead stress. Ecotoxicol. Environ. Saf..

[65] Kisa D., Kayır Ö., Sağlam N., Şahin S., Öztürk L., Elmastaş M. (2019). Changes of phenolic compounds in tomato associated with the heavy metal stress. Bartın Univ. Int. J. Nat. Appl. Sci..

[66] André C.M., Schafleitner R., Legay S., Lefèvre I., Aliaga C.A.A., Nomberto G., Hoffmann L., Hausman J., Larondelle Y., Evers D. (2009). Gene expression changes related to the production of phenolic compounds in potato tubers grown under drought stress. Phytochemistry.

[67] Ulhassan Z., Huang Q., Gill R.A., Ali S., Mwamba T.M., Ali B., Hina F., Zhou W. (2019). Protective mechanisms of melatonin against selenium toxicity in *Brassica napus*: Insights into physiological traits, thiol biosynthesis and antioxidant machinery. BMC Plant Biol..

[68] Jahan M.S., Guo S., Baloch A.R., Sun J., Shu S., Wang Y., Ahammed G.J., Kabir K., Roy R. (2020). Melatonin alleviates nickel phytotoxicity by improving photosynthesis, secondary metabolism and oxidative stress tolerance in tomato seedlings. Ecotoxicol. Environ. Saf..

